# Mechanical Performance of AlCrSiN and AlTiSiN Coatings on Inconel and Steel Substrates after Thermal Treatments

**DOI:** 10.3390/ma15238605

**Published:** 2022-12-02

**Authors:** Jing Liang, Eluxka Almandoz, Laia Ortiz-Membrado, Rafael Rodríguez, Jonathan Fernández de Ara, Gonzalo G. Fuentes, Luis Llanes, Emilio Jiménez-Piqué

**Affiliations:** 1 CIEFMA-Department of Materials Science and Engineering, EEBE, Universitat Politècnica de Catalunya-BarcelonaTECH, Avda. Eduard Maristany 16, 08019 Barcelona, Spain; 2 Centre of Advanced Surface Engineering, AIN, Cordovilla, 31191 Navarra, Spain; 3 Science Department, Universidad Pública de Navarra (UPNA), Campus de Arrosadía, 31006 Pamplona, Spain; 4 Institute for Advanced Materials and Mathematics (INAMAT2), Universidad Pública de Navarra (UPNA), Campus de Arrosadía, 31006 Pamplona, Spain; 5 Engineering Department, Public University of Navarre, Campus Arrosadía S/N, 31006 Pamplona, Spain; 6 Barcelona Research Center in Multiscale Science and Engineering-Universitat Politècnica de Catalunya-BarcelonaTECH, Avda. Eduard Maristany 16, 08019 Barcelona, Spain

**Keywords:** AlCrSiN, AlTiSiN, Inconel, nanoindentation, microscratch, thermal treatment, adhesion strength

## Abstract

The objective of this study was to explore the mechanical properties of AlCrSiN and AlTiSiN coatings deposited on Inconel and steel substrates after thermal treatments of 500 °C and 800 °C. Nanoindentation was used to measure the hardness and elastic modulus of the coatings, and microindentation was used for observing the contact damage with Hertzian contact loadings. Microscratch and Mercedes tests were used to evaluate the adhesive strength between coating and substrate with both progressive and static loads, respectively. The surface damage was inspected by optical microscopy and scanning electron microscopy (SEM). Focus ion beams (FIB) were used to mill the cross-sections in order to detect the extent and mode of failure. The results show that AlCrSiN coatings and Inconel substrates exhibit better mechanical performance, even after thermal treatments.

## 1. Introduction

Hard, protective coatings are frequently used in the tool industry due to their enhanced performance in wear and corrosion resistance. The first coatings used were TiN and CrN [1,2,3,4], but the incorporation in their composition of other elements, such as Al and Si, has resulted in new ternary coatings demonstrating increased performance [5,6,7]. For example, the introduction of Si produces an amorphous Si_3_N_4_ phase at the nanometer scale, which inhibits the sliding movement between neighboring grains [8] and enhances hardness and thermal stability [9,10]. However, tribocorrosion resistance is not optimal in these ternary coatings; therefore, the introduction of Al and Si to form quaternary coatings enhances the oxidation resistance and thermal stability of coatings via formation of an oxide-rich top layer [11,12,13]. Physical vapor deposition (PVD) for tool steel applied in various areas is a promising and effective technique to improve the mechanical performance and serving lives [14,15,16,17]. These quaternary coatings consist of a metastable amorphous phase which is crystalized after thermal treatment into an fcc/wurtzite lattice with high atomic density, forming a coating with a columnar structure and high hardness [18,19,20,21,22]. During operation, it is expected that these materials will suffer repetitive heating and cooling cycles with high temperatures reached in the coating of tool materials [23,24]. Therefore, it is important to understand the evolution of the mechanical properties of these coatings with thermal cycles, similar to those suffered in service. Understanding the evolution of mechanical properties has direct implications in enhancing the reliability of coated tool materials.

However, it is also important to understand that the performance of the tools is not only governed by the coating; the substrate also plays a relevant role in the deformation and fracture of the surface. Currently, most of the literature on mechanical performance of the quaternary coatings and multilayers only concentrates on the coating itself, ignoring the synergic effect with the substrate [25,26,27]. The tribological performance, oxidation resistance, and thermal stability of quaternary coating have been studied in several prior studies [28,29,30,31,32]; however, there is scarce information on the evolution of mechanical properties with thermal treatments. It is worth mentioning that those coating/substrate systems will undergo thermal cycles during operation, and it is relevant to understand the evolution of the mechanical properties under such cycles. In this sense, Liu et al. [28] observed the evolution of hardness after the thermal treatment of AlCrSiN and AlTiSiN coatings on stainless steel, reporting a decrease in the values after thermal treatment of 600 °C [21,33,34]. However, they did not report any adhesion testing.

In summary, there is not much information on the mechanical evolution of these systems with thermal treatments, and there are no reports on coating–substrate adhesion. Scarce information is reported about the evolution of mechanical performance after thermal treatments when AlCrSiN and AlTiSiN quaternary coatings deposited on two kinds of cutting tool substrates. In this sense, adhesion and fracture is a combination of the mechanical performance of both the coating and the substrate under these special conditions. Although these tool materials are designed to work at intermediate temperatures (500 °C), they may suffer occasional high temperature peaks at critical work points. At high temperature, the mechanical properties of the substrates also change. Both steel and Inconel suffer softening, due to grain growth and change in the topology of the precipitates [35,36]. In this sense, both types of materials usually require surface treatments to complement their bulk properties to yield good performance in service. Due to the different natures of their chemical composition, and thus, metallographic structure, the response of each material is different when depositing a PVD coating. Basically, the differences affect adhesion, densification (hardness), corrosion/oxidation resistance, roughness, etc.

The present study investigated the effect of thermal treatments on the mechanical properties of AlCrSiN and AlTiSiN coatings deposited on two different soft substrates, steel and Inconel, with the aim of providing a novel sight to elucidate the mechanical performance of quaternary coatings on engineering substrate system, with special focus on not only studying the mechanics of the coating, but also the coating/substrate interface.

In doing so, nanoindentation, microindentation, microscratch, and Mercedes tests were used to characterize the mechanical response of coatings.

## 2. Experimental Procedure

### 2.1. Sample Preparation

Two different coatings (AlCrSiN and AlTiSiN) were deposited on two substrates (steel and Inconel), making four different coating–substrate systems: AlCrSiN/steel, AlCrSiN/Inconel, AlTiSiN/steel, and AlTiSiN/Inconel. The steel substrate was H13 (DIN 1.2344). Additionally, the Inconel was an aged Inconel 718. The chemical compositions of the two substrates are presented in Table 1 and Table 2.

The coatings were produced by cathodic arc evaporation in a commercial METAPLAS MZR-323 PVD reactor able to reach a base pressure of 5·10^−^^4^ Pa. The reactor was equipped with two opposing columns, each of them hosting three circular cathodes (6.3 cm diameter) aligned vertically. Both columns faced each other and left an effective volume of 0.5 m^3^ available for the substrates. For the AlTiSiN coating, three cathodes of Ti (99.8% purity) and three cathodes of AlSi (80 at.% Al–20 at.% Si) were placed in an alternating arrangement (i.e., a Ti cathode faced an AlSi cathode). In the case of the AlCrSiN coating, the Ti cathodes were replaced by Cr cathodes (99.98% purity). The deposition parameter of the coatings is presented in Table 3 and the process temperature for both cases is 400–450 °C. The AlTiSiN coating had a Ti bonding layer and the AlCrSiN had a Cr + CrN bonding layer.

### 2.2. Coating Composition and Coating Thickness

Glow discharge optical emission spectroscopy (GDOES) was used to obtain the chemical composition of the coatings. The GDOES analyses were performed with a Jobin-Yvon JY 1000 RF optical spectrometer equipped with more than 40 channels and an optical monochromator. Coating thicknesses were measured by calowear tests, which were conducted specifically with a rotating a hard steel sphere of a known diameter to friction the surface of coating samples by continuously adding silica solution (30–50 weight percent) to increase the friction between sphere and samples. The solution may let the sphere abrade the coating and into the substrate, then form a spherical depression and seen from a plane the depression is rounded and followed Equation (1). By measuring the outer and inner edge radius of depression, the thickness of coating can be calculated as follows:

(1) $$t=\frac{\left(R+r\right)\left(R-r\right)}{d}$$

where *t* is the thickness of the coating (µm), *R* is the outer edge radius of depression, *r* is the inner edge radius of depression, and *d* is the diameter of the hard steel sphere.

### 2.3. Nanoindentation and Microindentation

The hardness and Young’s modulus of coatings were measured with an MTS Nanoindenter XP equipped with continuous stiffness measurement. Prior to nanoindentation, all samples were polished by colloidal silica and cleaned with acetone to reduce the effect of the roughness of coatings on the measurements [37]. Nanoindentation assays were performed with a Berkovich tip calibrated against a fused silica standard. A matrix of 25 imprints was derived for each sample at a constant strain rate of 0.05 s^−^^1^. The Oliver and Pharr method was used to calculate the hardness (*H*) and elastic modulus (*E*); the Poisson ratio was assumed to be *ν* = 0.25 [38]. Hardness was measured at 10% penetration depth and Young’s modulus of the coating was estimated by extrapolating the results to null thickness. The subscript *f* is used in the elastic modulus to indicate that it is the one of the coatings.

Micro contact damage tests were performed by Hertzian contact loading in a servo hydraulic test machine (Instron 8500) with a WC-Co sphere of 2.5 mm of diameter [39]. A trapezoidal wave was chosen as the loading curve with time, with a loading rate of 30 N/s rate, and maximum loads of 500 N or 750 N, held for 20 s.

Vickers tests on the substrates were performed with a Testwell FV-700 hardness tester under 10 kg load. The average and standard deviation for each sample under 10 kg load was obtained from five indentations.

### 2.4. Adhesion Test

Scratch tests and Mercedes tests were performed in order to measure the adhesion between coating and substrate. The reason for using two different tests was to induce different stress fields and damage scenarios at the interface by using different indenter tip geometries and loading conditions. Scratch tests were performed in a CSM Revetest with progressive loads from 0 to 30 N at a constant loading rate of 10 N/min with a Rockwell C diamond stylus of 200 μm radius and 120° apex angle, with a scratch length of 5 mm. Damage and failure were later observed by scanning electron microscopy (SEM).

To further characterize the contact damage of coated substrates, the adhesion was characterized by Mercedes test [40,41]. In this test, a Rockwell C intender was pressed against the surface of coated substrates, producing deformation and fracture. Four different loads were used: 98 N, 196 N, 392 N and 613 N in order to produce different amounts of damage.

### 2.5. Thermal Treatments

Two different temperatures were tested: 500 °C and 800 °C. Thermal treatments were conducted in an elevator furnace, starting from room temperature and heating at a 10 °C/min rate until the desired temperature. Samples were maintained at maximum temperature for 60 min, and then cooled down to room temperature.

### 2.6. Microscopy

In order to inspect the deformation and damage suffered by the coatings, Phenom XL SEM apparatus was used. The cross-section of the coatings was obtained with a Zeiss Neon 40 focus ion beam (FIB) [42], with a gallium source accelerated at 30 kV with a decreasing ion current down to a final polishing stage at 500 pA. To avoid the waterfall effect in the milling processing, a protective layer of platinum was deposited on the area of interest.

## 3. Results and Discussion

### 3.1. Coating Composition and Coating Thickness

**Figure 1:**
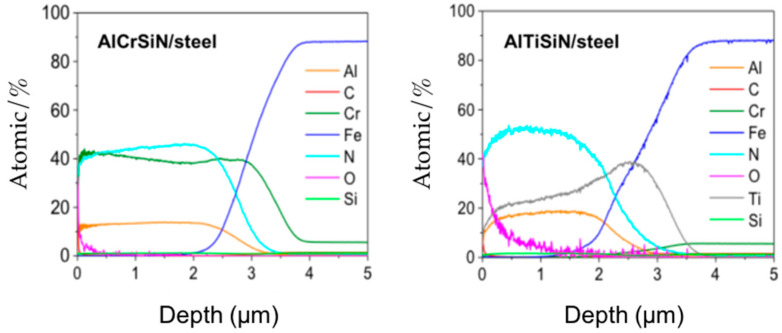
Composition (in atomic %) of the coatings vs. depth (µm) obtained by GDOES for AlCrSiN/steel (**left**) and AlTiSiN/steel (**right**).

The coating thicknesses measured by the calowear tests are presented in Table 4. While all values are around 2 µm, the thickness of the coatings deposited on steel is slightly thicker than the ones deposited on InconelReason for this may be attributed to the difference in the conductivity for the two substrates which results in different deposition rates. Composition of the coatings is presented in Figure 1.

### 3.2. Mechanical Properties of the Coated Materials

**Figure 2:**
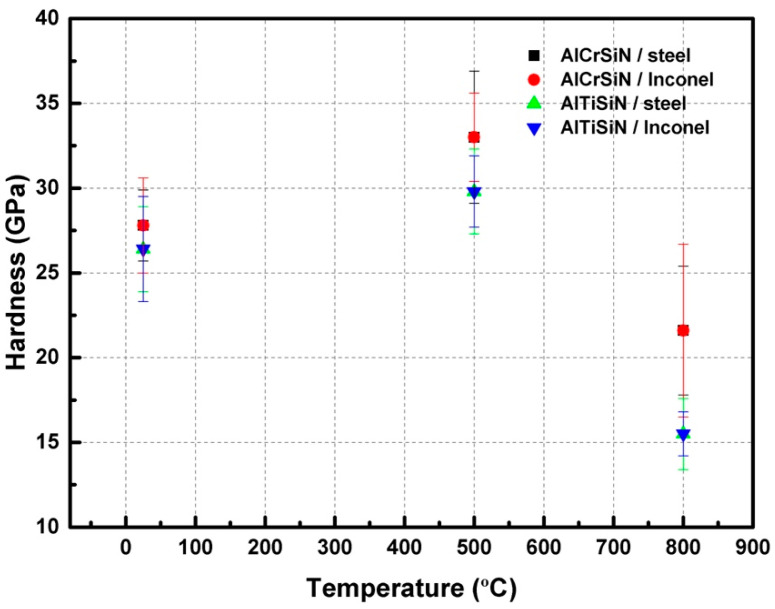
The relationship of the hardness of coatings with different thermal treatment.

**Figure 3:**
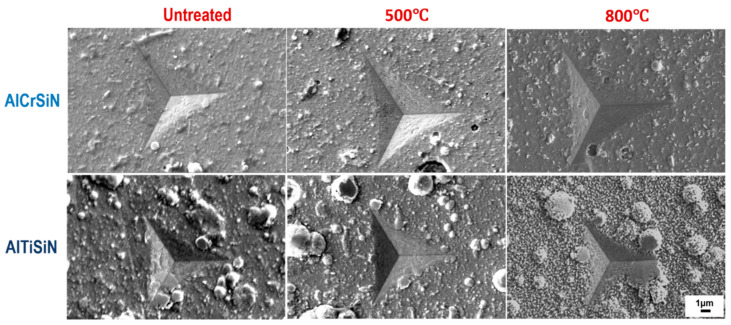
The SEM image of nanoindentation of AlCrSiN and AlTiSiN deposited on steel substrate after thermal treatment.

Nanoindentation results are presented in Table 5 and Figure 2. The images of the nanoindentation imprints are presented in Figure 3. The hardness and elastic modulus ratio (H/E) was adopted to describe elastic deformation to failure and H^3^/E^2^ is the plasticity index to present the resistance to plastic deformation. Both are common parameters to characterize the mechanical performance and wear resistance of coatings [43,44]. Based on Table 5, AlCrSiN coatings exhibited higher values of H^3^/E^2^ than AlTiSiN coatings when they all were deposited at the same substrate, indicating a better wear resistance response. It is elucidated that AlTiSiN coatings may present better wear resistance [45]. However, it should be considered that the performance of the tool materials is a combination of both the substrate and coating. In this respect, Vickers test results of the substrate are presented in Table 6.

It is seen how the samples that underwent a cycle of 500 °C presented an increase in the values of hardness, both for AlCrSiN and AlTiSiN coatings, regardless of the substrate. At 800 °C, hardness decreased. This may be attributed to the densification of the microstructure at moderate temperatures and the phase transformation at high temperatures where the cubic nitride phase transforms into a hexagonal nitrides phase [21,46].

Hardness of the substrates decreased in both cases, mainly after exposure at 800 °C. This is probably due to an increase in grain size and a modification of the precipitates [35,36].

Images of the indented coatings (Figure 3) present droplets at the surface typical of the PVD process [47]. The thermal treatment at 500 °C did not produce relevant changes in the surface. However, after the thermal treatment at 800 °C, there was a clear change in the surface, due to initial oxidation of the coatings. This oxidation is more evident in the case of the AlTiSiN coatings. This results in indentation imprints which deviates from the ideal shape, which gives rises to higher scattering in the mechanical values measured by this technique.

In Figure 3, it is also shown how, for AlCrSiN coatings, some ring cracks appear around the indentation, which may indicate a lower fracture toughness compared with AlTiSiN coatings [48].

### 3.3. Adhesion Tests

**Figure 4:**
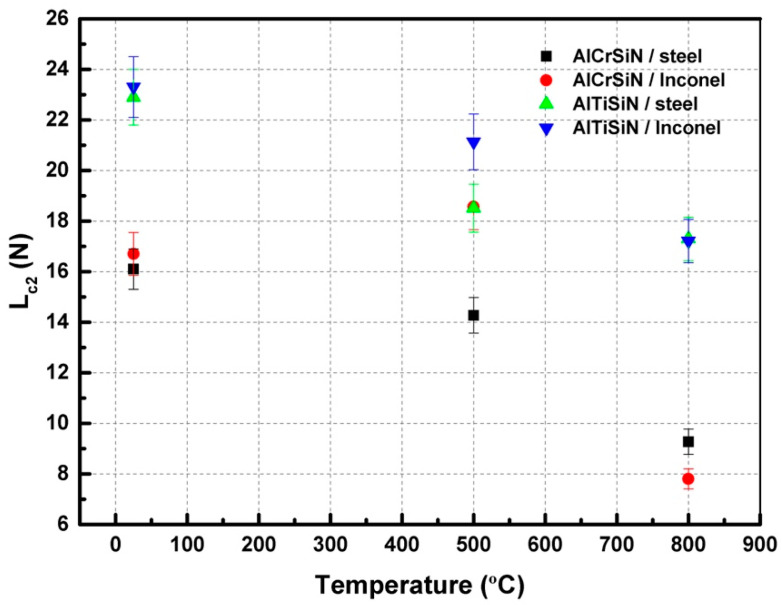
Critical load as a function of temperature of the thermal treatment.

Figure 4 presents the critical loads for delamination of all the materials. Figure 4 shows how the appearance of decohesion of the coating for the AlTiSiN coating is higher than AlCrSiN, independent of the substrate. However, after thermal treatments at 500 °C, the coatings deposited on Inconel presented a better adhesion than those deposited on steel. Furthermore, the AlCrSiN on Inconel coating thermally treated at 500 °C exhibited an enhanced scratch resistance compared with the samples without thermal treatment. This enhancement may be due to the higher thermomechanical stability of Inconel as compared with steel. For thermal treatments at 800 °C, all coatings present lower adhesion, due to degradation of the coating as well as softening of the substrates.

**Figure 5:**
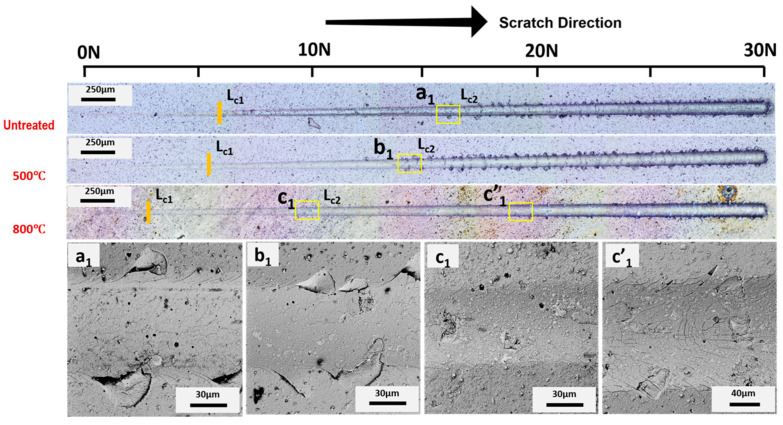
Adhesion optical profile of failure after microscratch tests of the AlCrSiN/steel sample. First (*L*_*c*1_) and second (*L*_*c*2_) critical loads are indicated. SEM magnification of selected areas (a_1_, b_1_, c_1_ and c’_1_) are also presented.

**Figure 6:**
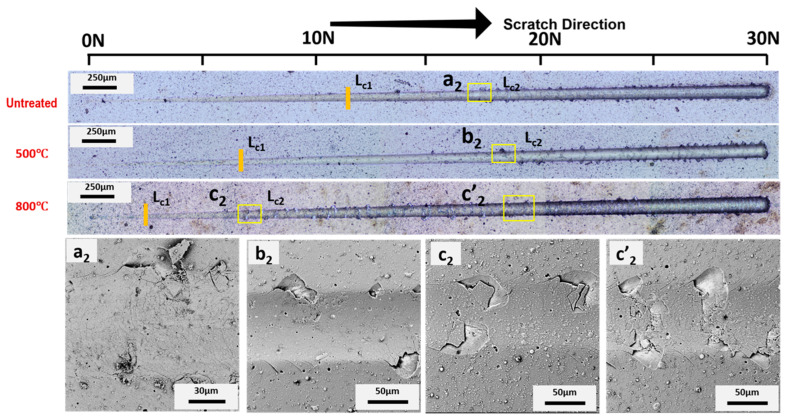
Adhesion optical profile of failures after microscratch tests of the AlCrSiN/Inconel sample. First (*L*_*c*1_) and second (*L*_*c*2_) critical loads are indicated. SEM magnification of selected areas (a_2_, b_2_, c_2_ and c’_2_) are also presented.

**Figure 7:**
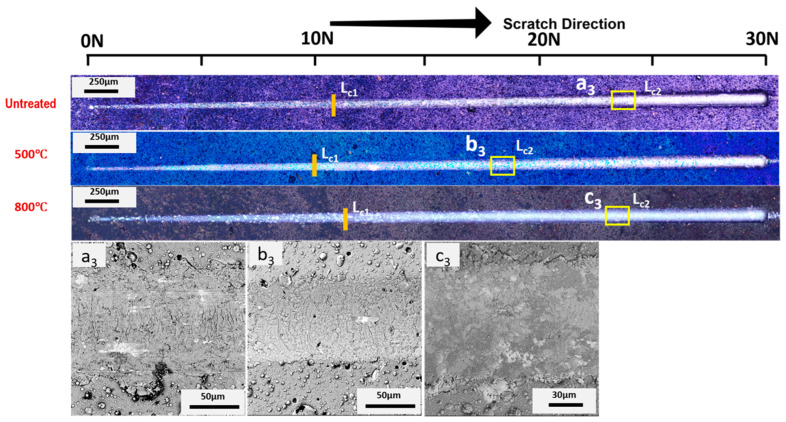
Adhesion optical profile of failure after microscratch tests of the AlTiSiN/steel sample. First (*L*_*c*1_) and second (*L*_*c*2_) critical loads are indicated. SEM magnification of selected areas (a_3_, b_3_ and c_3_) are also presented.

**Figure 8:**
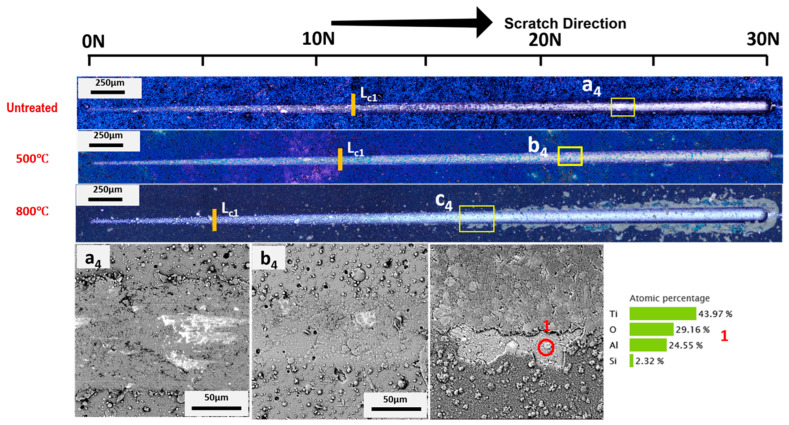
Adhesion optical profile and SEM images of failure and EDX analysis after microscratch tests of the AlTiSiN/Inconel sample. First (*L*_*c*1_) critical loads are indicated. SEM magnification of selected areas (a_4_, b_4_ and c_4_) are also presented as well as the chemical compostion by EDX of the indicated point.

Images obtained by optical microscopy; SEM of the scratch tracks are presented in Figure 5, Figure 6, Figure 7 and Figure 8 for AlCrSiN/steel, AlCrSiN/Inconel, AlTiSiN/steel, and AlTiSiN/Inconel, respectively. The coating thickness affects the scratch loads for adhesion and the direct comparison of performance by scratch with different coating/substrate system could not be taken in account. Therefore, both coatings have comparable thicknesses for every substrate. In Figure 5 and Figure 6, the scratch tracks of AlCrSiN on both substrates are presented. It is seen how plastic deformation, microcracking, and delamination are produced as load is increased. Stick–slip deformation induced by compressive stress appeared at the contour. External cracks in the scratch direction were formed as well. The load at which this failure appeared is labeled as critical load, *L*_*c*1_. As the load increased, transverse cracks appeared, induced by tensile stress, until the detachment of the coatings: this load is defined as *L*_*c*2_, which indicates failure of the interface of coated substrate.

After thermal treatment of the AlCrSiN coatings, critical loads were lower in the case of the steel substrate. In addition, the samples after thermal treatment at 800 °C presented a different type of damage, with more microcracking and less evident spalling. For the samples on the Inconel substrate, a slight improvement was appreciated for samples thermally treated at 500 °C, following the enhancement of mechanical properties observed previously. The mechanism for this enhancement may be the relaxation of the residual stress of AlCrSiN coating after 500 °C thermal treatment [49], whereas the decrease in adhesion may due to a phase transformation forming the fcc-CrN phase into h-Cr_2_N [50].

In the case of AlTiSiN coatings, as presented in Figure 7 and Figure 8, a small amount of deformation occurred at loads below 26 N [3]. As the loading increased, adhesive failure occurred for AlTiSiN coatings. The coatings spalled from the middle of the trace of the scratch. After thermal treatment, the amount of delamination diminished for both temperatures. The difference for AlTiSiN coatings deposited on Inconel after thermal treatment, especially at 800 °C, compared with steel as the substrate, is that part of coating was delaminated. A substantial area of the coatings peeled off. EDX was conducted at the delaminated area, showing that the elements were similar with AlTiSiN coatings, and indicating that a thin layer of the coating remained on the surface. Therefore, the failure may be considered cohesive failure and not coating delamination. The reason for this may be the fragile layer formed in the thermal treatment process as well as the evident softening of the substrate. This phenomenon could be explained by the phase transformation of fcc-AlN into hcp-AlN after 800 °C thermal treatment [51].

**Figure 9:**
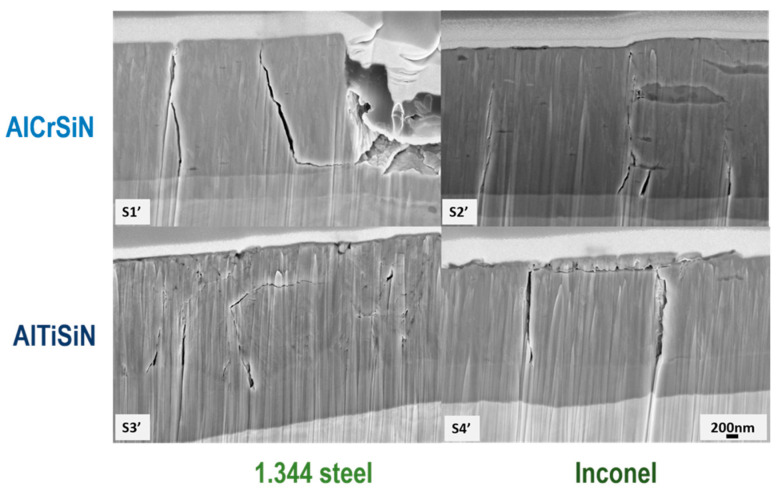
Cross-sectional SEM images at the same load (16 N) of scratch marked in Figure 5, Figure 6, Figure 7 and Figure 8. Labels S1’ to S4’ are included for better refence in Table 7.

In Figure 9, FIB cross-sections of all four coatings and microstructures are presented. The images were taken at the same load (16 N) in order to compare the behaviors between coatings. Figure 9 shows how that all cracks were arrested by interface, indicating a good structural integrity of the substrate. Both coatings had a dense microstructure and a bonding layer, as shown previously [26,52].

**Figure 10:**
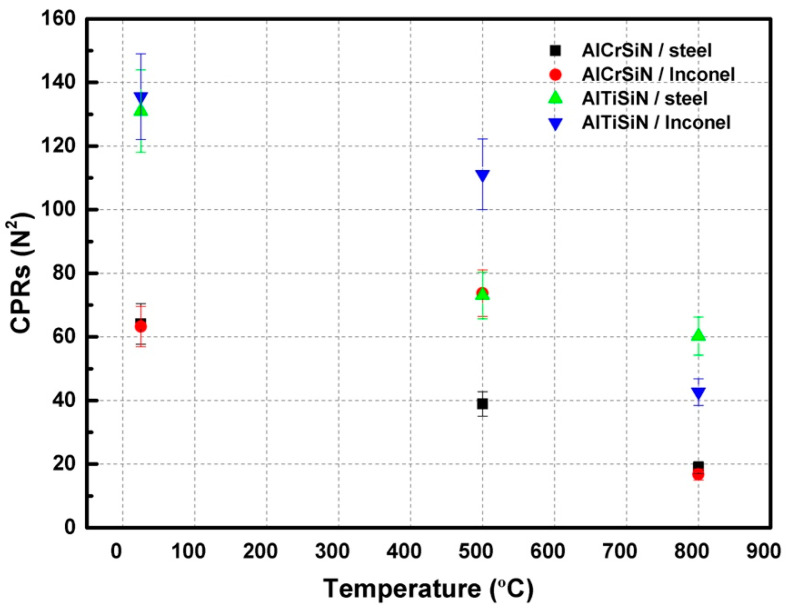
CPR of scratch tests after thermal treatment: AlCrSiN/steel, AlCrSiN/Inconel, AlTiSiN/steel, and AlTiSiN/Inconel.

In order to rationalize the scratch resistance of the coatings, the scratch crack propagation resistance (*CPR*) was calculated using Equation (2) [53].

(2) $$CPR=L_{c1}\left(L_{c2}-L_{c1}\right)$$

where *L*_*c*1_ is the critical load of start of lateral crack, and *L*_*c*2_ is the critical load of the start of delamination or spallation; the results are presented in Figure 10.

The critical stress *σ_c_* is calculated by,

(3) $$\sigma_{c}=\left(\frac{2L_{c2}}{\pi d_{c}^{2}}\right)\left[\frac{\left(4+\nu_{f}\right)3\pi \mu }{8}-1+2\nu_{f}\right)$$

where *d_c_* is the track width at *L*_*c*2_, *μ* is the friction coefficient calculated by the friction force, and *ν_f_* is the Poisson rate of coatings. The surface energy of the known interfacial crack is defined by Equation (4) [54,55,56]:

(4) Gc=σc2t2Ef

where *t* and *E_f_* are the thickness and the elastic modulus of coatings, respectively. The values of *E_f_* were experimentally determined from Table 5.

Crack propagation resistance (*CPR*) is presented in Figure 10, which shows how AlTiSiN coatings generally present higher *CPR* than AlCrSiN coatings, independently of the substrate. After thermal treatments, the *CPR* degraded in all cases, with the exception of AlCrSiN on Inconel, which presented a similar *CPR* after thermal treatment at 500 °C.

The AlTiSiN/Inconel sample presented the highest value of *G_c_* = 321.5 J/m^2^, as seen in Table 7. The range of values was similar to those previously report by other researchers for similar hard coatings [57,58,59]. Of all the studied systems, AlTiSiN/steel presented the higher adhesion energy. This coating also exhibited the highest *G_c_* after 800 °C thermal treatment. In comparison with AlTiSiN, AlCrSiN presented lower values for critical stress and adhesion energy, which was consistent with the analysis of the *CPR* microstratch test.

**Figure 11:**
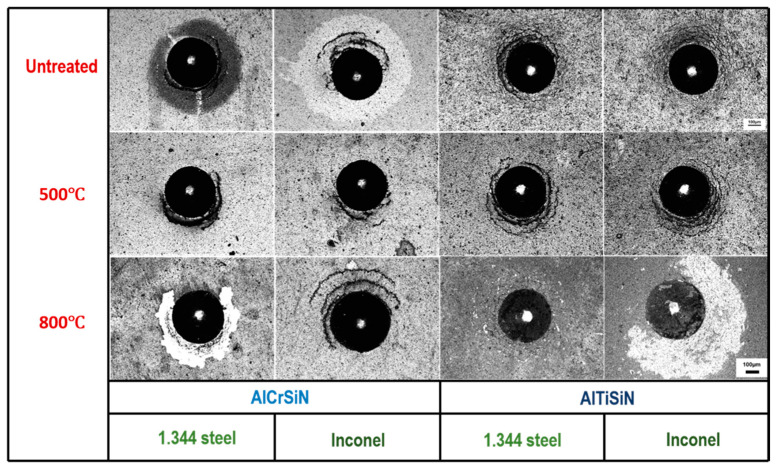
Confocal images of the Mercedes test at 613 N with a Rockwell C tip contrasted with different thermal treatments.

Figure 11 presents the results of the Mercedes Test at a normal load of 613 N. In all cases, radial cracks and partial ring cracks appeared. However, in the AlCrSiN/steel and AlTiSiN/Inconel systems after 800 °C thermal treatment, the vicinity of the indentation appeared with a high area of delamination. This high degree of delamination was also a consequence of the softening of the substrate, which resulted in higher deformation under contact loading and larger differential strains between the coating and substrate.

In the untreated AlCrSiN/Inconel sample, a similar phenomenon was noticed. In this case, delamination without spallation was observed, which indicated lower adhesion than after thermal treatment at 500 °C. This is coherent with the enhancement in CPR as observed in Figure 10.

**Figure 12:**
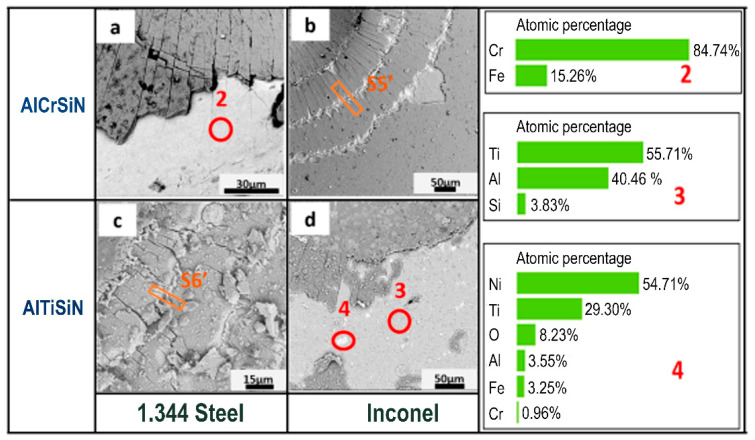
SEM images of the Mercedes test and EDX analysis at 613 N with a Rockwell C tip after 800 °C treatment: (**a**) AlCrSiN/steel, (**b**) AlCrSiN/Inconel, (**c**) AlTiSiN/steel, and (**d**) AlTiSiN/Inconel. Numbers 2m3 and 4 indicate the chemical analysis by EDS. S5’ and S6’ indicate the FIB cross-sections as presented in Figure 13.

**Figure 13:**
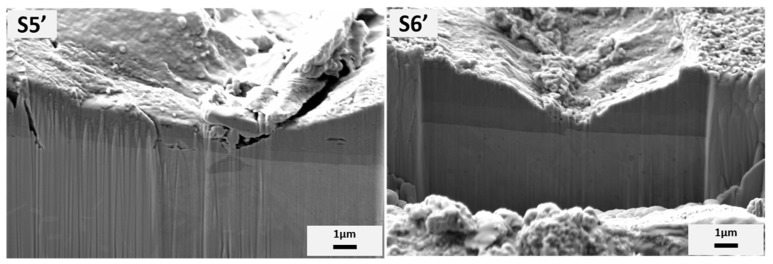
Cross-sectional SEM images with two magnifications of partial ring cracks at 613 N with a Rockwell C tip after 800 °C treatment marked in Figure 12.

In order to further explore the damage at 613 N after 800 °C, the particular area of indentation after Mercedes test was magnified by SEM, as shown in Figure 12, and EDX was performed to probe the elements of the delaminated area to ensure that the substrate was exposed. For AlCrSiN/steel, the exposed and light color area consisted of Cr and Fe. However, for AlTiSiN/Inconel, two contrasts appeared: shallow grey (circled as red 3) and white (circled as red 4). The SEM images demonstrate the delamination with large area of AlCrSiN/steel and AlTiSiN/Inconel at 613 N after 800 °C, but with traces of the coating still attached to the substrate. To further observe the internal deformation mechanisms, a cross-section at the areas was indicate as S5’and S6’, and is shown in Figure 13. In all cases, it was seen how the cracks were contained at the coating, without propagating into the substrate.

## 4. Conclusions

The mechanical properties of AlCrSiN and AlTiSiN quaternary coatings deposited on Inconel and steel which underwent thermal treatments at 500 °C and 800 °C have been characterized. The conclusions can be summarized as:

1. Nanoindentation results show values of hardness of 28 GPa and 26 GPa, and elastic modulus values of 369 GPa and 367 GPa for AlCrSiN and AlTiSiN coatings, respectively, before thermal treatment. After 500 °C, the hardness and elastic modulus increased for all samples. After the 800 °C thermal treatment, these properties decreased, indicating a degradation of the material.
2. Through microscratch tests, the AlTiSiN samples presented better adhesion and AlCrSiN coating displayed lower adhesion for all thermal treatments. This is further evidenced by the Mercedes test, where AlCrSiN coatings presented a higher detached area. Through SEM and FIB observations, it was evidenced that the mode of failure was cohesive.
3. Regarding the substrates, coatings deposited on Inconel exhibited better performance, especially after thermal treatment at 500 °C, associated with the better performance of Inconel at high temperature. Thermal treatments at 800 °C resulted in a degradation of the mechanical performance, due to the microstructural changes in both the coating and the substrate, such as increases in the grain size and modification of the precipitates. Based on the data of crack propagation resistance and surface energy of an interfacial crack, it is observed that the AlTiSiN coating presented better adhesion than the AlCrSiN coating, even after thermal treatments.

## Figures and Tables

**Table 1:** Composition (% by weight) of steel H13 (DIN 1.2344).

Composition (% by Weight)								
Name	C	Si	Mn	Cr	Mo	V	P	S
1.2344	0.37–0.43	0.90–1.20	0.30–0.50	4.80–5.50	1.20–1.50	0.90–1.10	0.030	0.030

**Table 2:** Composition (% by weight) of Inconel 718 used as a substrate.

Composition (% by Weight)							
Name	Ni	Cr	Nb	Mo	Ti	Al	Other Minor Elements
Inconel	50–55	17–21	4.75–5.50	2.80–3.30	0.65–1.15	0.2–0.8	Balence

**Table 3:** The deposition parameter of AlTiSiN and AlCrSiN coatings.

Coating	Step	Gas	Bias (V)	Total Pressure (mbar)	Rotating Speed (rpm)
AlTiSiN	Bonding layer	Ar	−60	1.2 × 10^−2^	5
	AlTiSiN deposition	N_2_	−60	4.0 × 10^−2^	5
	Bonding layer	Ar	−60	1.2 × 10^−2^	5
AlCrSiN		N_2_	−60	1.2 × 10^−2^	5
	AlCrSiN deposition	N_2_	−60	4.0 × 10^−2^	5

**Table 4:** Thickness of coated samples.

Coating	AlCrSiN		AlTiSiN	
Substrate	Steel	Inconel	Steel	Inconel
Thickness (µm)	2.9	1.6	2.6	1.7

**Table 5:** Mechanical properties of coated samples after thermal treatment.

Coating	AlCrSiN			AlTiSiN		
	Unheated	500 °C	800 °C	Unheated	500 °C	800 °C
Hardness (GPa)	28 ± 3	33 ± 4	22 ± 5	26 ± 3	30 ± 2	16 ± 2
Elastic Modulus (GPa)	369 ± 17	462 ± 46	280 ± 60	367 ± 42	476 ± 35	330 ± 16
HE	0.075	0.071	0.077	0.072	0.063	0.047
H3E2	0.158	0.168	0.128	0.137	0.117	0.034

**Table 6:** Vickers hardness of the substrates.

Sample	Untreated	500 °C	800 °C
Steel	476 ± 4	479 ± 2	417 ± 4
Inconel	461 ± 3	454 ± 3	316 ± 7

**Table 7:** Critical load and adhesion energy of the coated systems after thermal treatment.

No.	Sample		*G_c_* (J/m^2^)		
	Coating	Substrate	Untreated	500 °C	800 °C
S1’	AlCrSiN	Steel	72 ± 7	56 ± 6	30 ± 3
S2’	AlCrSiN	Inconel	87 ± 9	54 ± 5	23 ± 2
S3’	AlTiSiN	Steel	303 ± 30	105 ± 10	272 ± 27
S4’	AlTiSiN	Inconel	322 ± 31	162 ± 16	117 ± 10

## Data Availability

The data that support the findings of this study are available from the corresponding author upon reasonable request.
